# Band Gap Tuning in Transition Metal and Rare-Earth-Ion-Doped TiO_2_, CeO_2_, and SnO_2_ Nanoparticles

**DOI:** 10.3390/nano13010145

**Published:** 2022-12-28

**Authors:** Iliana Apostolova, Angel Apostolov, Julia Wesselinowa

**Affiliations:** 1 University of Forestry, Kl. Ohridsky Blvd. 10, 1756 Sofia, Bulgaria; 2 University of Architecture, Civil Engineering and Geodesy, Hristo Smirnenski Blvd. 1, 1046 Sofia, Bulgaria; 3 Sofia University “St. Kliment Ohridski”, J. Bouchier Blvd. 5, 1164 Sofia, Bulgaria

**Keywords:** TiO_2_, CeO_2_, SnO_2_ nanoparticles, ion doping, band-gap energy, s-d model

## Abstract

The energy gap $E_{g}$ between the valence and conduction bands is a key characteristic of semiconductors. Semiconductors, such as TiO${}_{2}$, SnO${}_{2}$, and CeO${}_{2}$ have a relatively wide band gap $E_{g}$ that only allows the material to absorb UV light. Using the s-d microscopic model and the Green’s function method, we have shown two possibilities to reduce the band-gap energy $E_{g}$—reducing the NP size and/or ion doping with transition metals (Co, Fe, Mn, and Cu) or rare earth (Sm, Tb, and Er) ions. Different strains appear that lead to changes in the exchange-interaction constants, and thus to a decrease in $E_{g}$. Moreover, the importance of the s-d interaction, which causes room-temperature ferromagnetism and band-gap energy tuning in dilute magnetic semiconductors, is shown. We tried to clarify some discrepancies in the experimental data.

## 1. Introduction

Dilute magnetic semiconductors (DMS)—bulk and nanostructures, doped with transition metal (TM) ions, have been intensively investigated in recent years due to the number of unusual electronic, magnetic, and optical properties. Recently, room-temperature ferromagnetism has been observed experimentally and theoretically in pure and ion-doped semiconducting TiO${}_{2}$, SnO${}_{2}$, and CeO${}_{2}$ nanostructures due to uncompensated spins or to oxygen vacancies at the surface and doping effects [1,2,3,4,5]. It is known that TiO${}_{2}$, SnO${}_{2}$, and CeO${}_{2}$ have a relatively wide band gap $E_{g}$ (∼3.2, ∼3.6, and ∼3 eV, respectively) [6,7,8], which limits their photocatalytic activity to the ultraviolet region of light. One effective way to overcome these problems is to modify the band gap of these compounds is doping with different elements, such as metals and nonmetals, which affect $E_{g}$ in different ways. Transition metals, such as Co, Fe, Ni, and Mn, lead to decreasing $E_{g}$ in TiO${}_{2}$, SnO${}_{2}$, and CeO${}_{2}$ NPs, as observed experimentally by many authors [9,10,11,12,13,14,15,16,17,18,19,20,21,22], improving the absorption of visible light. It must be mentioned that Salah et al. [23] and Venugopal et al. [24] observed an increase in $E_{g}$ in Mn-doped SnO${}_{2}$ NPs, i.e., there are some discrepancies.

In order to reduce the electron-hole recombination and to shift the absorption wavelength to the desired visible region (λ>400 nm), a decrease in the band gap of rare earth (RE)-doped TiO${}_{2}$, CeO${}_{2}$, and SnO${}_{2}$ bulk and nanoparticles (NPs) has been considered, which improves the absorption of visible light [25,26,27,28,29,30,31,32,33]. Let us emphasize that there are also results with enhanced $E_{g}$ by increasing the RE ion concentration [7,34]. Long et al. [33] obtained, in Sn-doped TiO${}_{2}$, a minimum of the band gap $E_{g}$ as a function of the doping concentration. The doping effects in Sn-doped TiO${}_{2}$ are reported to lead to the red-shift or blue-shift of $E_{g}$ [35,36] as well as in La-doped TiO${}_{2}$ [34,37]. Again, there are some discrepancies. First-principle studies are used to analyze the optical properties of TiO${}_{2}$, SnO${}_{2}$, and CeO${}_{2}$ doped with TM [38,39,40,41,42,43] and RE ions [44,45,46,47].

The aim of the present paper is using the s−d model to investigate on a microscopic level the band gap tuning of TM- and RE-doped TiO${}_{2}$, SnO${}_{2}$, and CeO${}_{2}$ NPs, which are promising candidates for applications of visible-light photocatalytic activities, and to explain the discrepancies reported in the experimental data of the band gap $E_{g}$ values. We will show that lattice deformations, lattice parameters, and surface and doping effects are strongly correlated with $E_{g}$ values.

## 2. Model and Method

The Hamiltonian, which describes the magnetic properties of TM and RE ion-doped DMS NPs, is the s-d(f) Hamiltonian proposed for ferromagnetic semiconductors [4]

(1) $$H_{s-d}=H_{sp}+H_{el}+H_{sp-el}.$$

$H_{sp}$ is the Heisenberg model of the d(f)-electrons in TM or RE metal ions

(2) $$H_{sp}=-\sum_{i,j}xJ_{ij}\left(S_{i}^{+}S_{j}^{-}+S_{i}^{-}S_{j}^{+}+2S_{i}^{z}S_{j}^{z}\right)-\sum_{i}D_{i}{\left(S_{i}^{z}\right)}^{2}.$$

Here, $S_{i}^{+}$, $S_{i}^{-}$, and $S_{i}^{z}$ are the spin-operators for the localized spins at site *i*, $J_{ij}$ is the magnetic exchange interaction between nearest neighboring sites *i* and *j*, $D_{i}$ is the single-site anisotropy parameter, and *x* is the ion-doping concentration.

The Hamiltonian of the conduction band electrons $H_{el}$ is observed after development according to Wannier functions and limited to one band taking into account interactions between different Wannier states [48]:

(3) $$H_{el}=\sum_{ij\sigma }t_{ij}c_{i\sigma }^{+}c_{j\sigma }+\frac{1}{2}\sum_{ijkl,\sigma \sigma^{\prime }}v\left(ijkl\right)c_{i\sigma }^{+}c_{j\sigma^{\prime }}^{+}c_{k\sigma^{\prime }}c_{l\sigma },$$

where $t_{ij}$ is the hopping integral, *v* is the Coulomb interaction, and $c_{i\sigma }^{+}$ and $c_{i\sigma }$ are the Fermi-creation and -annihilation operators.

The operator $H_{sp-el}$ couples the two subsystems—Equations (2) and (3)—by an intra-atomic exchange interaction $I_{i}$:

(4) $$H_{sp-el}=\sum_{i}xI_{i}S_{i}s_{i}.$$

The spin operators $s_{i}$ of the conduction electrons at site *i* can be expressed as $s_{i}^{+}=c_{i+}^{+}c_{i-}$, $s_{i}^{z}=\left(c_{i+}^{+}c_{i+}-c_{i-}^{+}c_{i-}\right)/2$. The important role of the s-d interaction *I*, taken into account in our paper, is supported by many experimental data. Room-temperature ferromagnetism may be due to sp−d exchange interactions (as observed in optical spectra for DMS), which may be responsible for the ferromagnetic properties of the samples [49]. The carriers are spin-polarized to mediate the ferromagnetic ordering of the magnetic moments of transition metal ions doped into the semiconductor host lattice. Moreover, for example, Kaushik et al. [50] have shown that the optical spectra have a red shift with increasing Co in TiO${}_{2}$ due to sp−d exchange interactions. The shift of $E_{g}$ in Mn-doped SnO${}_{2}$ NPs could also be attributed to the sp−d exchange interactions [51].

The band-gap energy $E_{g}$ is defined by the difference between the valence and conduction bands:

(5) $$E_{g}=\omega^{+}\left(k=0\right)-\omega^{-}\left(k=k_{\sigma }\right).$$

The electronic energies

(6) $$\omega^{\pm }\left(k\right)=\epsilon_{k}-\frac{\sigma }{2}I\langle S^{z}\rangle +\sum_{k^{\prime }}\left[v\left(o\right)-v\left(k-k^{\prime }\right)\right]\langle n_{k^{\prime }-\sigma }\rangle$$

are observed from the Green’s functions $g\left(k\sigma \right)=\ll c_{k\sigma };c_{k\sigma }^{+}\gg$, σ=±1. $\langle n_{k^{\prime }\sigma }\rangle$ is the occupation number distribution. $\langle S^{z}\rangle$ is the magnetization calculated in our previous work [4].

For the approximate calculation of the Green’s functions g(kσ), we use a method proposed by Tserkovnikov [52]. After a formal integration of the equation of motion for $g_{ij}$, one obtains

(7) $$g_{ij}\left(t\right)=-i\theta \left(t\right)\langle \left[c_{i}^{-};c_{j}^{+}\right]\rangle \exp\left(-i\omega_{ij}\left(t\right)t\right)$$

where

(8) $$\begin{array}{ccc} \omega_{ij}\left(t\right)=\omega_{ij} & - & \frac{i}{t}\int_{0}^{t}dt^{\prime }t^{\prime }\left(\frac{\langle \left[j_{i}\left(t\right);j_{j}^{+}\left(t^{\prime }\right)\right]\rangle }{\langle \left[c_{i}^{-}\left(t\right);c_{j}^{+}\left(t^{\prime }\right)\right]\rangle }\right.\\ & - & \left.\frac{\langle \left[j_{i}\left(t\right);c_{j}^{+}\left(t^{\prime }\right)\right]\rangle \langle \left[c_{i}^{-}\left(t\right);j_{j}^{+}\left(t^{\prime }\right)\right]\rangle }{{\langle \left[c_{i}^{-}\left(t\right);c_{j}^{+}\left(t^{\prime }\right)\right]\rangle }^{2}}\right) \end{array}$$

with the notation $j_{i}\left(t\right)=\langle \left[c_{i}^{-},H_{interaction}\right]\rangle$. The time-independent term

(9) $$\omega_{ij}=\frac{\langle \left[\left[c_{i}^{-},H\right];c_{j}^{+}\right]\rangle }{\langle \left[c_{i}^{-};c_{j}^{+}\right]\rangle }$$

is the spin excitation energy in the generalized Hartree–Fock approximation. The time-dependent term in Equation (8) includes damping effects.

## 3. Results and Discussion

For the numerical calculations of ion-doped TiO${}_{2}$, we have taken the following model parameters: *J* = 0.31 meV, *D* = −0.1 meV, *I* = 60 meV, *v* = 0.3 meV, *F* = 4 cm${}^{-1}$, *R* = −0.35 cm${}^{-1}$, *J*(Co-Co) = 44 meV [53], *J*(Cu-Cu) = 30 meV [54], *J*(Fe-Fe) = 36.5 meV [55], *J*(Sm-Sm) = 0.3 meV [5], and *J*(Tb-Tb) = 1.5 meV [56], *J*(Er-Er) = 6 meV [57].

For example, the TiO${}_{2}$ NP has a spherical shape and the Ti spins are situated into shells numbered by n=1,...,N, where n=1 is the central spin and n=N—the surface shell, due to the changed number of next neighbors on the surface and to the fact that the reduced symmetry $J_{s}$ on the surface is different from that in the bulk $J_{b}$, as well as due to different strains $J_{d}$ in the doped states that are different to the undoped ones $J_{b}$. Moreover, *J* is inverse-proportional to the lattice parameters. 

### 3.1. Size Dependence of the Band-Gap Energy

**Figure 1:**
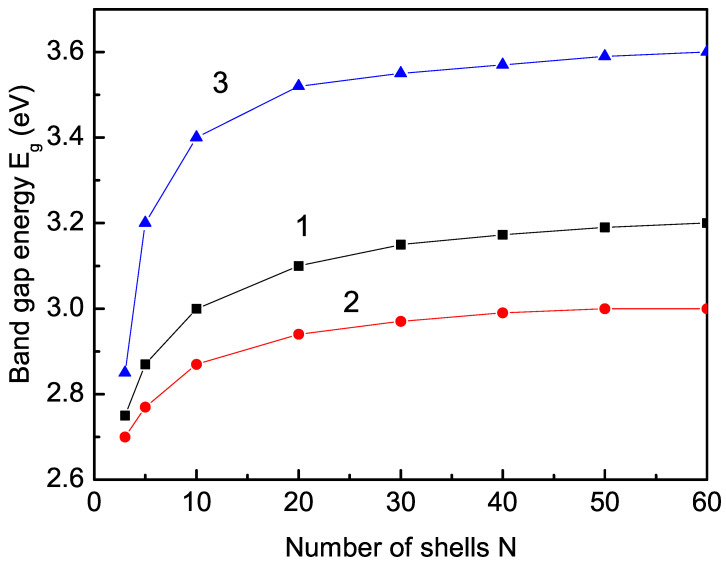
Size dependence of the band-gap energy $E_{g}$ for $J_{s}=0.8J_{b}$, *T* = 300 K for a (1) TiO${}_{2}$, (2) CeO${}_{2}$, and (3) SnO${}_{2}$ NP.

The first way to narrow the band gap $E_{g}$ of TiO${}_{2}$, SnO${}_{2}$, and CeO${}_{2}$ is to reduce the size of the pure undoped compound. In general, in TiO${}_{2}$ the interaction between the Ti${}^{4+}$ ions (*S* = 0) is diamagnetic. However, on the surface of non-doped TiO${}_{2}$ NPs due to uncompensated spins, there Ti${}^{3+}$ ions appear with S≠0, and thus magnetism is induced. Santara et al. [58] have observed that the reduction in the particle size leads to an increase in the lattice parameter, i.e., to an increase in tensile strain. In our model, this would lead to a decrease in the exchange interactions $J_{s}$ on the surface compared to the bulk value $J_{b}$, i.e., $J_{s}<J_{b}$. In Figure 1, curve 1, the size dependence of $E_{g}$ for TiO${}_{2}$ is shown. It can be seen that $E_{g}$ decreases with decreasing NP size, as reported experimentally by Chen et al. [29], Kalathil et al. [59], Garcia et al. [60], and Dette et al. [61]. This decrease in $E_{g}$ with the decrease in NP size is valid for all three compounds and is shown in Figure 1, curves 2 and 3, for CeO${}_{2}$ and SnO${}_{2}$, respectively, in coincidence with the experimental data for CeO${}_{2}$ NPs of Ansari et al. [8] and Tatar et al. [62] but not of Tamizhdurai et al. [63], as well as for SnO${}_{2}$ NPs of Kamarulzaman et al. [64], but in disagreement with the result of Asaithambi et al. [65], who reported an increase in $E_{g}$ with the decrease in the size of SnO${}_{2}$ NPs.

### 3.2. Ion-Doping Dependence of the Band-Gap Energy

#### 3.2.1. Ion-Doping Dependence of the Band-Edge Energies

**Figure 2:**
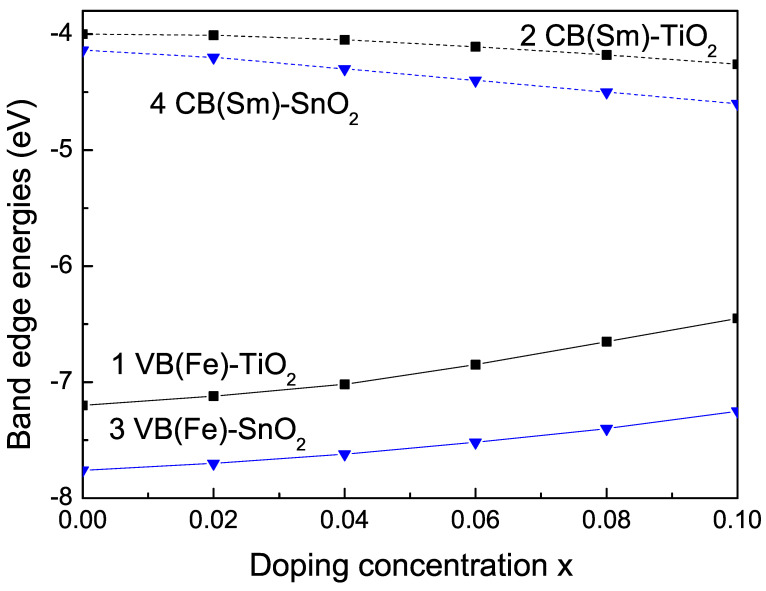
Dependence of the (1,3) VB edge energy of Fe-doped and (2,4) of the CB edge energy of Sm-doped bulk TiO${}_{2}$ and SnO${}_{2}$, respectively, on the ion-doping concentration *x*.

The conduction band (CB) in TiO${}_{2}$ is dominated by the empty Ti *d*-band, whereas the valence band (VB) is composed of the occupied O *p*-band and Ti *d*-band. Fujisawa et al. [66] and Dorenbos [67] have observed the valence and conduction band energies in bulk TiO${}_{2}$ to be VB = −7.25 (−7.2) eV and CB = −4.05 (−4.0) eV, respectively. For SnO${}_{2}$, the reported values are for VB = −7.76 [67] (−8.2 eV [68]) and for CB = −4.14 [67] (−4.6 eV [68]). However, it must be noted that the data are not consistent. Ion doping can move the band edges of the VB and/or the CB. Next, we will study the influence of the doping with a TM, for example, Fe, and an RE ion, for example, Sm, on the VB and CB energies in bulk TiO${}_{2}$ and SnO${}_{2}$. The transition metal ions form a dopant level above the VB band of TiO${}_{2}$ [10,37,69]. Fe${}^{3+}$ is known to create shallow trapping sites at the donor and acceptor levels [37]. This leads to a decrease in the band gap of Fe-doped TiO${}_{2}$. In Figure 2, curve 1, the calculation from Equation (6), using the s-d model, VB energy as a function of the Fe concentration, is presented. It can be seen that there is an increase in the VB edge energy. This increase is due to the contribution from the lower Fe 3d band, which benefits the hole mobility in VB. As a result of Fe-doping, the electron transition energy from the VB to the CB decreases, which may induce a red shift at the edge of the optical-absorption range. A broadening of the VB in Fe-doped TiO${}_{2}$ is reported by Wu et al. [11]. The incorporation of RE ions, for example, Sm, into the TiO${}_{2}$ host modifies the band gap of TiO${}_{2}$ with sub-band-gap energy levels of RE ions under the CB. These energy levels offer an electronic transition from the TiO${}_{2}$ valence band to the empty RE ion sub-band-gap energy levels. From Figure 2, curve 2, a reduction in the calculated CB edge energy in bulk Sm-doped TiO${}_{2}$ can be seen, as observed experimentally by Wei et al. [70], too. We also observed similar behavior for the dependence of the VB and CB energies of Fe (curve 3) and Sm (curve 4) doped bulk SnO${}_{2}$.

#### 3.2.2. Transition-Metal-Ion (Co, Fe, Mn, and Cu) Doping Effect on the Band-Gap Energy

**Figure 3:**
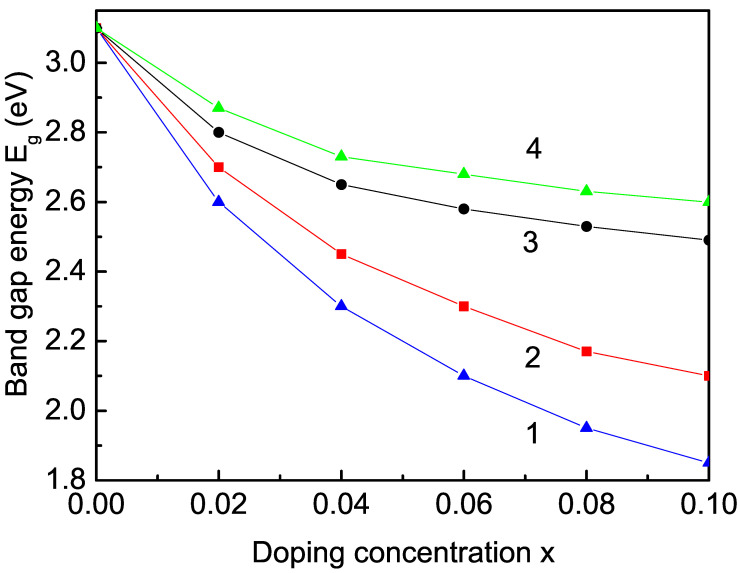
Dependence of the band-gap energy $E_{g}$ for *T* = 300 K on the ion-doping concentration *x* in a TiO${}_{2}$ NP (*N* = 20 shells) for (1) Co, (2) Fe, (3) Mn, and (4) Cu.

Next, we will study the band-gap energy $E_{g}$ in a TM-doped TiO${}_{2}$ NP, Ti${}_{1-x}$TM${}_{x}$O${}_{2}$, for example, with Fe. $E_{g}$ is calculated with our model from Equation (5). The difference of the ionic radius of Fe${}^{3+}$ (0.64 $\dot{A}$) to that of Ti${}^{4+}$ (0.68 $\dot{A}$) [71] means that the doped ion has a smaller ionic radius than the host ion, i.e., compressive strain appears. Our calculations show a decrease in the band-gap energy $E_{g}$ with increasing Fe-doping concentration *x* in a TiO${}_{2}$ NP (see Figure 3, curve 2), which is in agreement with other results [37,38,41,43,72]. The photochemical studies of George et al. [9] showed that band-gap energy $E_{g}$ in Fe-doped TiO${}_{2}$ was reciprocally tuned proportional to the Fe content. Shortly, we will discuss the importance of the s-d interaction. It must be noted that for simplification we only consider the *s* electrons and the s−d interaction. Due to the compressive strain, the exchange-interaction constants between the Ti${}^{3}$ and the Ti${}^{3+}$-Fe${}^{3+}$ ions lead to a small magnetization, which increases with increasing Fe-doping concentration. In order to explain the data strong decrease in the band gap by TM ion doping observed in the experimental data, we must take the large s-d interaction (see Equation (6)) into account, which strongly reduces the band-gap energy $E_{g}$.

In Figure 3 (curves 1 and 4), the band gap reduction in TiO${}_{2}$ NP with other TM ions is demonstrated, for example, with Co${}^{2+}$ (0.745 $\dot{A}$) [50] and Cu${}^{2+}$ (0.87 $\dot{A}$) [65], whose ionic radii are larger than the radius of the host Ti ion, i.e., unlike in the Fe ion doping, tensile strain appears. We have to use the relation $J_{d}<J_{b}$. This means that the exchange interaction between the Ti${}^{3+}$ ions in the doped states would decrease compared to the undoped ones, and we would observe a small magnetization *M* that would slowly decrease with increasing Co or Cu dopants. It must be noted that the double exchange interaction energy between these TM ions and the Ti${}^{3+}$ ion is ferromagnetic and stronger than that between the Ti${}^{3+}$-Ti${}^{3+}$ ions; together with the strong s−d interaction, it can change the behavior of the magnetization *M*, *M*, and it can increase when the concentration *x* is raised. Thus, we again obtain a decrease in the band-gap energy $E_{g}$ as observed in [10,50,73,74,75]. Let us emphasize that similar behavior is also reported in TM-doped CeO${}_{2}$ [13,72,76,77,78] and SnO${}_{2}$ NPs [12,65,79]. In order to clarify the discrepancies by Mn ion doping, let us emphasize that, with our model, we obtain a reduced band gap in a Mn${}^{2+}$ (*r* = 0.8 $\dot{A}$)-doped SnO${}_{2}$ NP using *J*(Mn-Mn) = 20.24 meV [12] (see Figure 3, curve 3). The ionic radius of the doped Mn${}^{2+}$ ion is smaller than that of the host Sn${}^{4+}$ ion (0.83 $\dot{A}$), i.e., again there appears a compressive strain; we have $J_{d}>J_{b}$. The substitution of Sn${}^{4+}$ by Mn${}^{2+}$ would require the formation of oxygen vacancies for charge balance, which is important for the RTFM and the band-gap reducing. It is advantageous to the photocatalytic activity, too [80]. Our result is in good qualitative agreement with that of Chatterjee et al. [81] for Mn-doped CeO${}_{2}$ NPs, as well as with the behavior in Mn-doped TiO${}_{2}$ [17,37] and Mn-doped SnO${}_{2}$ NPs [14,82], but in disagreement with Refs. [23,24,83], which reported an increase in the band-gap energy $E_{g}$ in Mn-doped SnO${}_{2}$ NPs despite the observed compressive strain. In our opinion, these discrepancies are due to the experimental methodology of synthesis and of growth, to the method of doping, and to the method of annealing.

#### 3.2.3. Rare Earth (Sm, Tb, and Er) Ion Doping Effects on the Band-Gap Energy

**Figure 4:**
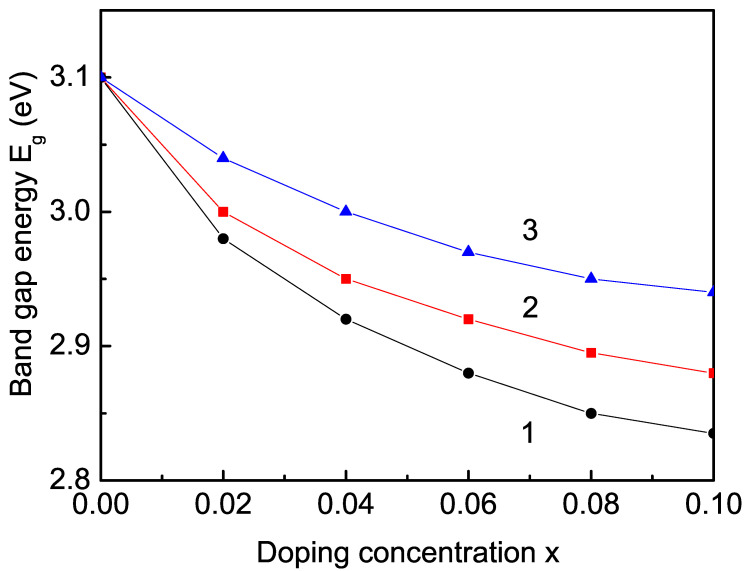
Dependence of the band-gap energy $E_{g}$ for *T* = 300 K on the ion-doping concentration *x* in a TiO${}_{2}$ NP (*N* = 20 shells) for (1) Sm, (2) Tb, and (3) Er.

Next, we will study the observed decrease in the band-gap energy $E_{g}$ in a RE (for example, Sm, Tb, and Er)-doped TiO${}_{2}$, Ti${}_{1-x}$RE${}_{x}$O${}_{2}$ NP. The ionic radius of Ti${}^{4+}$ is smaller than that of Sm${}^{3+}$ = 1.09 $\dot{A}$, and it is smaller than the ionic radius of the most RE ions. This means that there is a tensile strain. In Figure 4, we have calculated the band-gap energy $E_{g}$ as a function of the ion-doping concentration for different RE ions. It can be seen that $E_{g}$ decreases with increasing *x*. This is due to the reduction in the CB edge energy (see Figure 2, curve 2), reported also by Wei et al. [70]. Let us emphasize that the role of the intra-atomic s−d exchange interaction is very important here. The situation is in analogy with the doping with the TM ions Co and Cu. The tensile strain leads to changes in the exchange-interaction constants $J_{d}$ between the Ti${}^{3+}$ ions in the doped states and the undoped ones $J_{b}$, i.e., we have to use $J_{d}<J_{b}$. It must be noted that if we only take this interaction into account, then we will observe a small decrease in the magnetization [5] and of the band gap $E_{g}$. However, taking into account the strong s−d interaction and the interaction between Ti and the doping ion, the magnetization *M* changes its behavior, and it increases with increasing doping concentration *x*. An increase in *M* with increasing Sm and Nd ion doping is reported in [84]. Therefore, the band-gap energy $E_{g}$ decreases (see Figure 4, curve 1) in coincidence with [70,85] but not with [20]. The reduction in the band-gap energy of Sm-doped TiO${}_{2}$ indicates a red shift of the light adsorption. A decrease in $E_{g}$ in Sm-doped CeO${}_{2}$ NPs is also observed in [86,87,88], which can increase the photocatalytic activity. The decrease in $E_{g}$ with increasing Tb (*r* = 1.06 $\dot{A}$) or Er (*r* = 1.03 $\dot{A}$) doping concentration is presented in Figure 4, curves 2 and 3, in coincidence with Lee et al. for Er-doped TiO${}_{2}$ thin films [89]. We obtain within our s−d model a decrease in $E_{g}$ in TiO${}_{2}$, CeO${}_{2}$, and SnO${}_{2}$ with the most RE ions. Moreover, the band-gap energy $E_{g}$ decreases with the increase in the s−d interaction *I*. Let us emphasize that there are some discrepancies by the RE ion doping. A decrease in $E_{g}$ for small doping concentrations of different RE ions (for example, Sm, La, Dy, Nd, Eu, Er, and Tb) is also reported in TiO${}_{2}$, CeO${}_{2}$, or SnO${}_{2}$ NPs [70,85,90,91,92,93,94]. However, some authors have observed an increase in the band-gap energy $E_{g}$ in Sm [95] and Yb, Sc [45]-doped TiO${}_{2}$ NPs, and Pr-doped CeO${}_{2}$ NPs [96].

**Figure 5:**
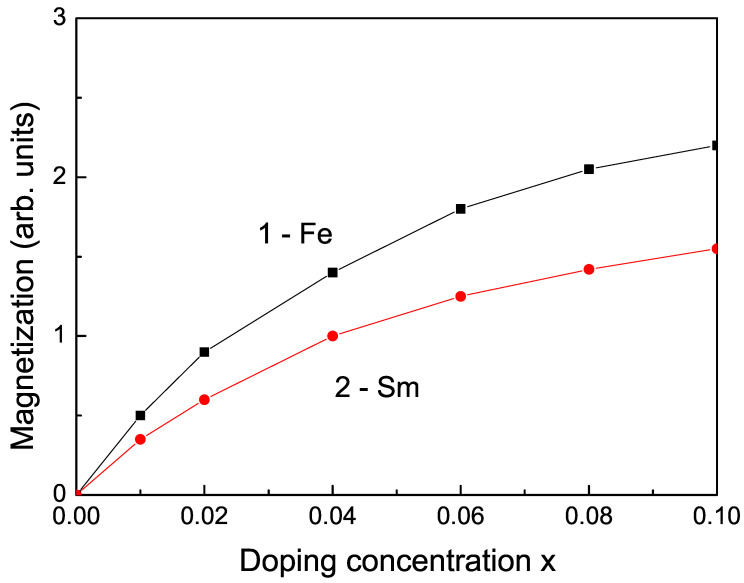
Dependence of the magnetization *M* on the ion-doping concentration (1) Fe and (2) Sm for $J_{s}=1.2J_{b}$, *T* = 300 K, for bulk TiO${}_{2}$.

**Figure 6:**
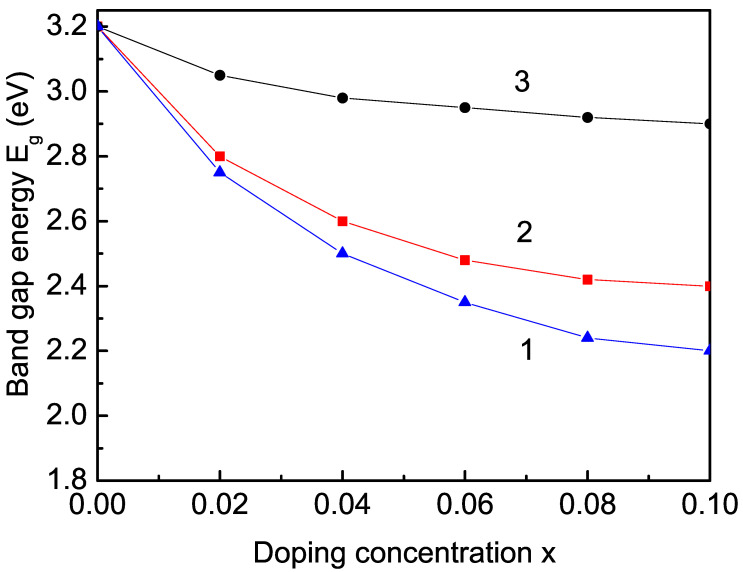
Dependence of the band-gap energy $E_{g}$ on the doping concentration *x* in bulk TiO${}_{2}$: (1) Co, (2) Fe, and (3) Sm, for $J_{s}=1.2J_{b}$, *T* = 300 K.

It must be mentioned that pure bulk TiO${}_{2}$ without defects and impurities is diamagnetic due to the presence of Ti${}^{4+}$, which has no unpaired electrons, making it diamagnetic. Doping TiO${}_{2}$ with TM or RE ions in order to induce unpaired spins introduces the required magnetic ordering to this compound. Due to the exchange interactions between the substituted doping ions on the Ti sites, there appears a magnetic moment, a spontaneous magnetization *M* different from zero, which increases with the increase in the doping concentration *x* (see Figure 5). The observed RTFM in ion-doped bulk TiO${}_{2}$ is reported in [43,46,69]. Moreover, $E_{g}$ in TM- and RE-doped bulk TiO${}_{2}$ decreases (see Figure 6) in good qualitative agreement with the experimental data [11,38,43]. This is also valid for the other two compounds CeO${}_{2}$ and SnO${}_{2}$.

## 4. Conclusions

In conclusion, narrowing the optical band gap of TiO${}_{2}$, SnO${}_{2}$, and CeO${}_{2}$ NPs is essential for visible light applications. Using the s−d microscopic model, we have shown on a microscopic level two possibilities to reduce the band-gap energy $E_{g}$ of CeO${}_{2}$, TiO${}_{2}$, or SnO${}_{2}$ NPs, reducing the NP size and/or ion doping with TM (Co, Fe, Mn, and Cu) or RE (Sm, Tb, and Er) ions, which can improve the photocatalytic activity. Different strains appear due to the different ionic radii of the TM or RE ions and the host ions, which lead to lattice deformations. Furthermore, the discrepancies by the $E_{g}$ behavior in Mn-doped and RE-doped TiO${}_{2}$, CeO${}_{2}$, and SnO${}_{2}$ NPs are discussed. We have shown that the decrease in the band-gap energy $E_{g}$ by RE doping is much smaller than that by TM doping. Therefore, one can conclude that the doping with TM ions is a more effective method than the doping with RE ions in order to reduce the band width $E_{g}$ in CeO${}_{2}$, TiO${}_{2}$, and SnO${}_{2}$ NPs and thus to enhance the photocatalytic activity.

## Data Availability

The raw data that support the findings of this study are available from the corresponding author upon reasonable request.
